# Teaching of Non-Clinical Skills should be embedded throughout Medical Training

**DOI:** 10.15694/mep.2018.0000187.1

**Published:** 2018-09-04

**Authors:** Sarah Procter, Jane Sturgess

**Affiliations:** 1 Mid Essex Hospitals NHS Trust; 2 West Suffolk NHS Trust

**Keywords:** Non-clinical skills, training, quality improvement, management, leadership

## Abstract

This article was migrated. The article was marked as recommended.

Medical leadership has been a focus of criticism after a number of notable health service failures. In order to address the development of a safe and accountable service for the future, medical trainees should be engaged in learning and participating in medical management from an early stage. We discuss measures we have taken at one hospital in the United Kingdom to embed non-clinical skills training in the natural progression of specialist training. This programme has since been expanded to other hospitals. We aim to disseminate our learning further so that others may consider implementing local training in a similar way.

## The Current Context

A series of high profile failings in healthcare delivery from 2005 onwards has led to some challenging reports for leaders to digest (Department of Health 2012, Public Inquiry 2013). Many of the findings have centred on dysfunctional medical leadership and poor attitudes to quality improvement and change. As such, emphasis is shifting to the delivery of a safe, high quality, patient-centric service - as reflected in The NHS Next Stage Review ( Department of Health 2008) and The Future Hospital Commission (Royal College of Physicians, 2013).

Much of the focus of medical training is rightly on the acquisition of clinical skills, yet the Royal Colleges’ curricula (for example, Royal College of Anaesthetists, 2010) include sections on professionalism and non-clinical skills that address issues covered in the reports. A number of factors adversely affect trainee engagement in this essential area of development, such as short hospital rotations, exam fixation, and a lack of understanding of what exactly is required by both trainee and trainer. At present, training in non-clinical skills is largely left to the discretion of trainees with inconsistencies across specialties and healthcare providers in both teaching and assessment. It is often an afterthought, addressed solely to satisfy the criteria for advanced training sign offs and consultant applications.

To provide some better guidance, the Faculty for Medical Leadership and Management have produced Developing Medical Leadership. A Toolkit for Doctors in Postgraduate Training ( FMLM, 2018), yet the onus remains with the trainee.

Where educational opportunities exist, they are typically polarised into generic short courses or exclusive fellowships and residencies. The former may not be sufficient in isolation, and the latter are limited by the commitment in time or funding - scarce resources for trainees. A FMLM survey (2018) of 400 junior doctors highlighted the educational gap that exists, with 97% of trainees recognising the importance of non-clinical skills training to them and future healthcare provision, yet 50% feeling their training was inadequate and 20% not knowing where to seek help. Whilst many (83%) had performed an audit, only 31% had seen their project become sustainably implemented within their place of work. This raises a number of questions: Is audit being performed merely to satisfy revalidation requirements rather than to deliver a genuine quality improvement project and act as a foundation for leadership and management training? How can trainers engage trainees in quality improvement, and maintain their morale, when the projects they perform are of such little value that they are not embedded into working practice? The survey makes clear the urgent need for trusts and departments to supply local teaching to bridge this gap, ideally with minimal additional cost to trainees and organisations.

## Our Model for Delivery of Training

Our department has developed a project to improve the quality of medical leadership and management training with activities of direct relevance to the consultant role. It consists of:

1. A framework of suggested projects and courses across the levels of training
2. An internal series of free lunchtime educational meetings for all trainees led by members of our local senior management, complimented with social media feeds and groups
3. A designated consultant lead for non-clinical skills

As a first step, we developed a framework of demonstrable targets that could provide evidence to reflect the curriculum requirements, with increasing depth of complexity and learning as trainees progress through the levels of training. These targets are work-place based, related to delivery of care, contextual, meaningful and not too onerous for either the trainee or the trainer. Many can be achieved naturally within the day-to-day work of the trainee. The aim of the framework was to promote the learning that occurs within even the smallest project to a conscious level and prompt the trainee to keep records of training in a formalised way. A future development could be a separate Professionalism Exercise (similar to other workplace based assessments) to enable trainees to discuss and document a non-clinical topic.

Our second step was to institute a series of in-house sessions with healthcare leaders, from Trust Board members including the Chief Executive to representatives from Health Education England. The series runs on a rolling basis and has been designed to cover core management topics. It is a cross specialty programme that opens up local management to all trainees regardless of specialty or training stage. We have chosen an hour at lunchtime, once a month on alternating weekdays, in a location at the heart of the hospital, in an effort to be as inclusive as possible. Multispecialty training offers a real insight into challenges faced by others, facilitates networking and provides an opportunity to discuss meaningful change whilst recognising it does not occur by involving one department alone. Each session has been evaluated and the cumulative trainee response since the start of the programme has been positive.

Many departments have lead clinicians for governance, research and equipment with a remit of administration and engagement of the department as a whole. As a third step in our project, we designated a lead clinician for non-clinical skills training. We advocate one in each trust, and preferably each department. Leads should have good knowledge on local, regional and national projects that are suitable and available for trainee involvement, and an understanding of quality improvement methodology and leadership credibility. Whether or not this individual should be a consultant is open to debate. The lead should guide trainees to choose realistic activities, appropriate to their stage of training, whilst encouraging them to develop an area of interest. Many trainees will be inclined to specialise in one particular non-clinical role such as teacher, manager or researcher. The role of the lead will be to make explicit the overlap that naturally occurs, and the skills that are learnt. For example, a ‘teacher’ may learn management and organisation skills whilst arranging and embedding an educational intervention into a teaching programme, and may learning quality improvement methodology either by assessing and evaluating the project, or identifying ways to overcome barriers to successful implementation. In this way, the trainee can deliver something that they can engage with and can contribute meaningfully to the development of safe healthcare without undertaking excess workloads. Allowing trainees to observe and participate in more challenging management processes, such as serious untoward incident investigation and business planning, rather than excluding them can also equip the next generation of clinicians to become part of the solution.

Mentorship can be provided by expert non-medical members of the healthcare delivery team, or clinical leaders from a different parent specialty, with feedback to the trainee’s overall educational supervisor. The development of a multi-disciplinary faculty of supervisors to oversee the education and application of non-clinical skills is the natural next step in delivering leadership and management training; our faculty delivering the in-house sessions has been made up entirely of senior trust leaders, distributing their expertise with no need for faculty training.

## Conclusions

In order to produce a generation of doctors who are engaged in medical leadership, safety and improvement, the culture of training has to be shifted to inclusive training for all from an early stage with spiral learning as trainees progress. It should be endemic and accessible in every trust, reducing the need to attend external courses to meet learning needs.

Our programme will continuously evolve with the curriculum and portfolio platforms, and with national directives for the delivery of leadership and management training. Our principle objective remains to comprehensively cover core non-clinical skills in a way that is accessible to all trainees.

## Take Home Messages

• Departments should provide more opportunities for trainees to be involved in meaningful local projects
• Increased on site training in non-clinical skills should reduce dependence on external training
• Non-clinical skills training should be easily available for all

## Notes On Contributors

Dr Sturgess is Associate Dean for Health Education East of England and a Consultant Anaesthetist.

Dr Procter is an anaesthetic trainee with a special interest in non-clinical skills training.
