# Supplementary material for: Teaching of Non-Clinical Skills should be embedded throughout Medical Training
Source: MedEdPublish (2016). 2018 Sep 4;7:187. [Version 1] doi: 10.15694/mep.2018.0000187.1 (PMC10701856; doi:10.15694/mep.2018.0000187.1)
Supplement: Peer review history

# Reviewer report: Reviewer response for version 1

**DOI:** 10.21956/mep.19649.r29299

**Published:** 2018-09-20

**Authors:** Wouter A. Keijser

**Affiliations:** 1 University of twente

This review has been migrated. The reviewer awarded 4 stars out of 5 Timely, UK-based, concise case report also reflecting need for more structured, field-based, hence local initiatives on non-tech skills for physicians. Keep them coming. Thanks.

Reviewer Expertise:

NA

No decision status is available

# Reviewer report: Reviewer response for version 1

**DOI:** 10.21956/mep.19649.r29300

**Published:** 2018-09-05

**Authors:** Gert Van Zyl

**Affiliations:** 1 University of the Free State

This review has been migrated. The reviewer awarded 4 stars out of 5 A very important discussion in the bigger discussion of non-clinical skills and leadership. More discussion would have contributed to the debate on the matter. I take it that the first take home message is linked to having projects available to teach and learn these non-clinical skills described. Please continue with the research and report on the findings.

Reviewer Expertise:

NA

No decision status is available

# Reviewer report: Reviewer response for version 1

**DOI:** 10.21956/mep.19649.r29298

**Published:** 2018-09-04

**Authors:** Trevor Gibbs

**Affiliations:** 1 AMEE

This review has been migrated. The reviewer awarded 3 stars out of 5 An interesting paper to read. Although, as the authors state, this relates to a UK based organisation, but I would expect the issue of leadership and the development of non-clinical skills applies to most training programmes in nay country.I realise that this is a personal opinion piece but I feel that it covers a useful area in healthcare training and the way that this organisation has organised their training is very structured and very clear, and demonstrates a very good example. The references are particularly useful to anyone working in the area.My only reservation was I wanted to know more- I wanted to read more of the content of the programme and how it was received by the participants over a short and an extended period of time. Seeing these in the paper would have certainly raised the star rating. I hope that the authors follow this project up and provide those important elements and I wish them well in their very essential project.

Reviewer Expertise:

NA

No decision status is available
